# Ferromagnetism and semiconducting of boron nanowires

**DOI:** 10.1186/1556-276X-7-678

**Published:** 2012-12-17

**Authors:** Jiling L Li, Tao He, Guowei Yang

**Affiliations:** 1 State Key Laboratory of Optoelectronic Materials and Technologies, Institute of Optoelectronic and Functional Composite Materials, Nanotechnology Research Center, School of Physics and Engineering, Sun Yat-sen University, Guangzhou 510275, Guangdong, People’s Republic of China

**Keywords:** Boron nanowires, Ferromagnetism, Semiconducting

## Abstract

More recently, motivated by extensively technical applications of carbon nanostructures, there is a growing interest in exploring novel non-carbon nanostructures. As the nearest neighbor of carbon in the periodic table, boron has exceptional properties of low volatility and high melting point and is stronger than steel, harder than corundum, and lighter than aluminum. Boron nanostructures thus are expected to have broad applications in various circumstances. In this contribution, we have performed a systematical study of the stability and electronic and magnetic properties of boron nanowires using the spin-polarized density functional calculations. Our calculations have revealed that there are six stable configurations of boron nanowires obtained by growing along different base vectors from the unit cell of the bulk α-rhombohedral boron (α-B) and β-rhombohedral boron (β-B). Well known, the boron bulk is usually metallic without magnetism. However, theoretical results about the magnetic and electronic properties showed that, whether for the α-B-based or the β-B-based nanowires, their magnetism is dependent on the growing direction. When the boron nanowires grow along the base vector [001], they exhibit ferromagnetism and have the magnetic moments of 1.98 and 2.62 μ_B_, respectively, for the α-c [001] and β-c [001] directions. Electronically, when the boron nanowire grows along the α-c [001] direction, it shows semiconducting and has the direct bandgap of 0.19 eV. These results showed that boron nanowires possess the unique direction dependence of the magnetic and semiconducting behaviors, which are distinctly different from that of the bulk boron. Therefore, these theoretical findings would bring boron nanowires to have many promising applications that are novel for the boron bulk.

## Background

Boron is very special in the periodic table as the nearest neighbor of carbon and has exceptional properties of low volatility, high melting point, stronger than steel, harder than corundum, and lighter than aluminum. Hence, studies on boron nanostructures have become more and more attractive in the recent years [1-13]. Among them, boron one-dimensional nanostructures are expected to have broad applications for their high conductivity, high aspect ratios, and excellent performance in harsh conditions [14-20]. In the last several years, so many experimental studies have performed on the one-dimensional boron nanowires, and a great progress has been obtained up to now [21-27]. Just recently, the vertically aligned single-crystalline boron nanowire arrays have been especially prepared [21]. Therefore, further explorations theoretically and experimentally on the one-dimensional boron nanostructures appear to be timely and desirable. However, the possible configurations and stability, as well as the electronic and magnetic properties of boron nanowires, which are important for the experimental preparation and technological applications, have not been reported so far. As a result of the well-aligned single-crystalline boron nanowires reported [21], in this contribution, we perform a theoretical study on the stability and magnetic and electronic properties of boron nanowires growing from the unit cells of stable B bulks.

## Methods

Herein, we firstly get the different boron nanowires from the growth of the unit cell of the bulk boron, respectively, along different base vectors. Well known among the various boron allotropes, the most stable phases of the boron bulk are the α-rhombohedral (α-B) and β-rhombohedral (β-B) boron [28]. The α-B is the simplest one that consists of a distorted B_12_ icosahedron per unit cell, forming an fcc-like structure. The β-B is the most commonly found modification and can be considered as an fcc-like structure consisting of the B_84_ quasi-spheres together with the B_10_-B-B_10_ chains located in the octahedral interstices formed by the B_84_ spheres [29]. In the following study, we respectively attain three different boron nanowires from the growth of the unit cell of the ground states of α-B and β-B along different base vectors. We then carry out the first-principles investigation of the stability and electronic and magnetic behaviors of the considered boron nanowires. Additionally, the dependence of the electronic and magnetic properties on the growth direction of boron nanowires is discussed. These investigations are expected to provide valuable information for future applications of boron nanostructures.

We perform the first-principles spin-polarized density functional theory (DFT) using the SIESTA computation code [30-32], which is based on the standard Kohn-Sham self-consistent DFT. A flexible linear combination of numerical atomic-orbital basis sets is used for the description of valence electrons, and norm-conserving nonlocal pseudopotentials were adopted for the atomic cores. The pseudopotentials are constructed using the Trouiller-Martins scheme [33] to describe the interaction of valence electrons with atomic cores. The nonlocal components of pseudopotential are expressed in the fully separable form of Kleiman and Bylander [34,35]. The Perdew-Burkle-Ernzerhof form generalized gradient approximation corrections are adopted for the exchange-correction potential [36]. The atomic orbital set employed throughout is a double-ζ plus polarization function. The numerical integrals are performed and projected on a real space grid with an equivalent cutoff of 120 Ry for calculating the self-consistent Hamiltonian matrix elements. For boron nanowires under study, periodic boundary condition along the wire axis is employed with a lateral vacuum region larger than 25 Å to avoid the image interactions. The supercell of boron nanowires respectively contains one unit cell of α-B and β-B as translational unit growing along different directions. To determine the equilibrium configurations of these boron nanowires, we relax all atomic coordinates involved using a conjugate gradient algorithm until the maximum atomic force of less than 0.02 eV/Å is achieved. In the calculations of the total energies and the energy band structures, we use four *k* sampling points along the tube axis according to the Monkhorst-Pack approximation. Cohesive energy (*E*_*c*_) is calculated according to the expression, *E*_*c*_ *=* (*E*_total_ *− n* × *E*_*B*_) / *n*, where *E*_total_ is the total energy of the considered boron nanowire, *n* is the number of B atoms, and *E*_*B*_ is the energy of an isolated B atom.

## Results and discussion

**Figure 1:**
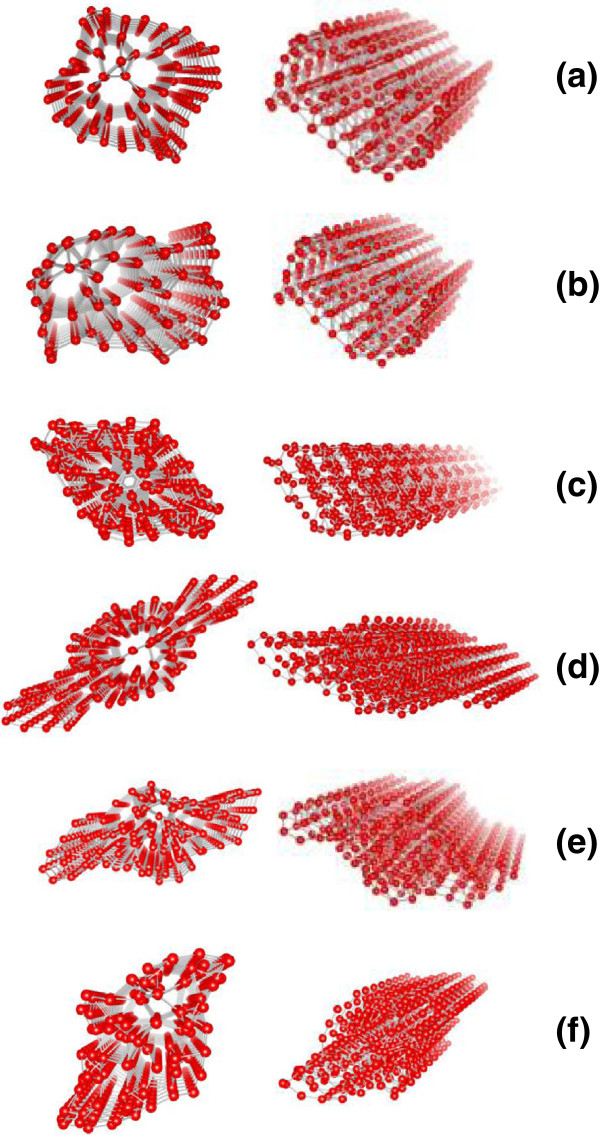
**Optimized configurations of the considered boron nanowires (red circles).** (**a**) α-a [100]_,_ (**b**) α-b [010], (**c**) α-c [001], (**d**) β-a [100], (**e**) β-b [010], and (**f**) β-c [001]. Herein, for the same configuration, the left and right are respectively corresponding to the side and top views.

Firstly, we construct the stable configurations of the bulk α-B and β-B. The optimized configurations in the present study keep the same perfect structure as previously proposed [28,29]. Also, according to the structural characteristic of the bulk α-B and β-B, in the following study, six possible representative nanowires are considered. Three were obtained from the unit cell of α-B, growing along three base vectors, respectively. The other three were from the unit cell of β-B, also growing respectively along the base vectors. The corresponding boron nanowires are denoted according to the based bulk boron and their growth direction, named by α-a [100], α-b [010], α-c [001], β-a [100], β-b [010], and β-c [001]. For all these constructed boron nanowires, we perform a complete geometry optimization including spin polarization. Their equilibrium configurations are respectively shown in Figure 1a,b,c,d,e,f, where the left and right are respectively the side and top views for the same configuration. These results thus reveal that the optimized configurations of the six under-considered boron nanowires still keep the same perfect B-B bond structure as those in the bulk boron. To evaluate the stability of these boron nanowires, we calculate their cohesive energies by determining the cohesive energies according to the definition discussed previously. The calculated cohesive energies are listed in the first column of Table 1. For comparison, in Table 1, we also give the cohesive energies calculated at the same theoretical level of the bulk α-B and β-B. A negative cohesive energy value indicates that the chemical energies processed to form the boron nanowires are exothermic in reaction. The cohesive energies of all the considered boron nanowires are negative and have the absolute value larger than 6.70 eV/atom. This indicates that the dispersive B atoms prefer to bind together and form novel nanostructures, which can be seen from literatures about the multi-shaped one-dimensional nanowires [21-27]. Simultaneously, by comparison, the cohesive energies of the considered boron nanowires are a little smaller than those of the bulk α-B and β-B, which are the two most stable of the various B bulks. Therefore, we conclude that all these under-considered boron nanowires are chemically stable. However, due to the relatively higher cohesive energy, some of the considered boron nanowires may be metastable, and experimental researchers need to seek the path of synthesizing these materials. Nevertheless, the typical one-dimensional structural characteristic and the attractive electronic and magnetic properties, as shown below, may stimulate experimental efforts in searching for a synthesizing path of this material. 

**Table 1:** Cohesive energies and total magnetic moments of considered boron nanowires and of bulk α-B and β-B

**Nanostructure**	**E**_***c ***_**(eV/atom)**	**M (μ**_**B**_**)**
α-a [100]	−6.88	0.02
α-b [010]	−6.94	0.00
α-c [001]	−6.84	1.98
β-a [100]	−6.75	0.00
β-b [010]	−6.74	0.00
β-c [001]	−6.76	2.62
α-B	−7.42	0.00
β-B	−7.39	0.00

To lend further understanding of the nature of the boron nanowires considered above, we studied the electronic structures of all configurations using the spin-polarized calculations. The calculated total magnetic moments of the six nanowires are listed in the second column of Table 1. It is obvious that for the three boron nanowires obtained from the unit cell of α-B, the nanowires α-a [100] and α-b [010] have the total magnetic moments of approximately equal to zero, while the nanowire α-c [001] has a distinctly different total magnetic moment of 1.98 μ_B_. Moreover, for the three boron nanowires obtained from the unit cell of β-B, the same trend about the total magnetic moments occurs. The nanowires β-a [100] and α-b [010] both have the total magnetic moments also approximately equal to zero, and the nanowire β-c [001] has the total magnetic moments of 2.62 μ_B_. Additionally, in Table 1, we also presented the calculated total magnetic moments of bulk α-B and β-B. Thus, these results indicate that both of the two kinds of boron bulks have no magnetism, with the total magnetic moments equal to zero.

**Figure 2:**
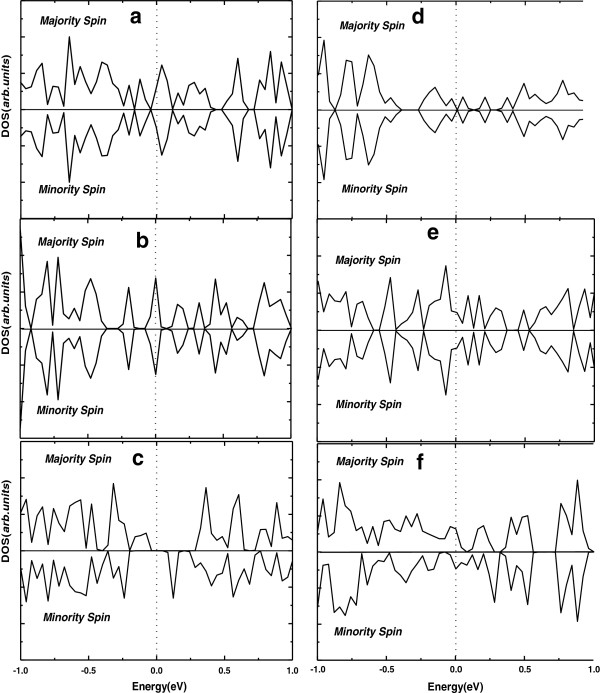
**PDOS of the considered systems.** (**a**) α-a [100], (**b**) α-b [010], (**c**) α-c [001], (**d**) β-a [100], (**e**) β-b [010], and (**f**) β-c [001]. Positive and negative values represent the DOSs projected on the spin up and down, respectively. The Fermi levels are denoted by the vertical dashed line.

For the two magnetic nanowires, α-c [001] and β-c [001], we also set the initial spin configurations to the antiferromagnetic (AFM) order. The difference of the total energy between AFM and ferromagnetic (FM) (Δ*E* = *E*_AFM_ − *E*_FM_) is 0.031 and 0.100 eV, respectively, corresponding to nanowires α-c [001] and β-c [001]. This result indicates that both of the two magnetic nanowires are in the FM ground state. To lend further understanding about magnetic properties of the considered boron nanowires, we calculate the projected total electronic density of states for all considered boron nanowires, as plotted in Figure 2. Clearly, we can see that for both of the two magnetic nanowires, the majority (spin-up) state and minority (spin-down) state are not compensated, which resulted in the residue of net spin states, as seen in Figure 2c,f. However, as shown in Figure 2a,d,e,f, the other boron nanowires are spin-compensated, with the spin-up and spin-down states equally occupied.

**Figure 3:**
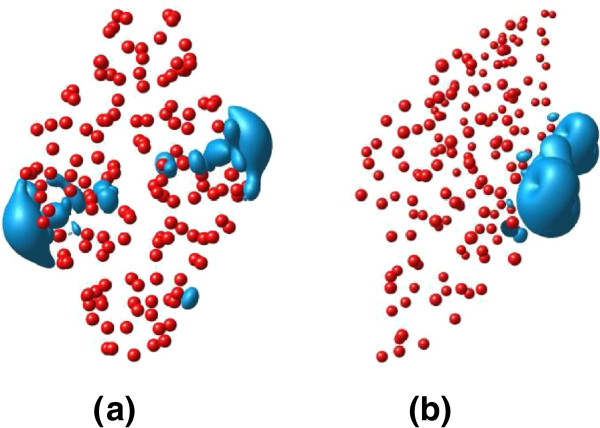
**The isosurface of spin density *ρ*** **=** ***ρ***^**↑**^ **−** ***ρ***^**↓ **^**of the supercells of the two magnetic boron nanowires (red circles).** (**a**) α-c [001] and (**b**) β-c [001]. The isovalue is set to 0.30 e/Å^3^.

To pursue the physical origin of the magnetic moments of the two magnetic boron nanowires, we plot the isosurface of spin density of the supercells of the two magnetic boron nanowires, respectively, as shown in Figure 3a,b. The isovalue is set to 0.30 e/Å^3^. It thus is obvious that for the boron nanowire α-c [001], the total magnetic moment of the system is essentially contributed from the atoms near two vertexes of one diagonals of the cross section. The spin density is symmetrically distributed around the two ends of the diagonals. For the boron nanowire β-c [001], the spin density is mainly distributed near one vertex of the diagonals in the cross section, which is in agreement with the previous report [37]. The key to understand why the magnetic boron nanowires have the magnetic moments around the vertexes of one diagonals of the cross section is the atomic structural characteristic and especially the structural deformation of the magnetic boron nanowires tailored from the bulk boron. By analyzing, we find out that the reasons of the induced magnetic moments are mainly from two aspects. One is the unsaturated chemical bonds of the atoms at the vertexes of the diagonal, which make the electron states redistributed and cause the asymmetry of the spin-up and spin-down states. Another aspect is the local magnetic moments around the ends of the diagonal act by the interaction of spin-spin coupling, which enhances the total magnetic moments of the two magnetic boron nanowires and makes them show distinct and much larger total magnetic moments. 

**Figure 4:**
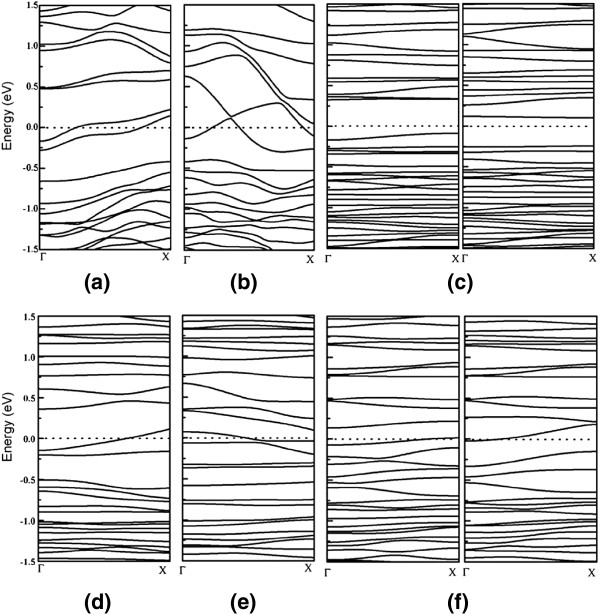
**The band structures near the Fermi level.** (**a**) α-a [100]_,_ (**b**) α-b [010], (**c**) α-c [001], (**d**) β-a [100], (**e**) β-b [010], and (**f**) β-c [001]. For (c) and (f), the left and right respectively represent the bands of spin-up and spin-down electrons. The dashed lines represent the Fermi level *E*_F._

To complete the description of the study on boron nanowires, it is important to analyze their electronic properties of all configurations. The electronic energy band structure of the considered boron nanowires are shown in Figure 4, in which the Fermi levels are denoted by the dashed line in this figure. Herein, for boron nanowires having no magnetic moments, we recalculated the band structure by performing DFT without spin polarization, as shown in Figure 4a,b,d,e. While for both of the two magnetic nanowires, we give the band structures calculated using the spin-polarized DFT. The calculated band energy structures are shown in Figure 4c,f, wherein the left and right respectively represent the bands of spin-up and spin-down electron states. Clearly, we can see that most of the boron nanowires under study are metallic with the electronic energy bands across the *E*_F_, as shown in Figure 4. However, as seen in Figure 4c, the band structure of the boron nanowire α-c [001] is obviously different from that of the other metallic nanowires. In detail, the boron nanowire α-c [001] is a narrow bandgap semiconductor with a direct energy gap of 0.19 eV at X point. Due to the well-known shortcoming of DFT in describing the excited states, DFT calculations are always used to understand the bandgaps of materials. Therefore, the bandgap value, 0.19 eV, obtained from the present calculations may be underestimated. However, this value clearly indicates that the electronic property of the boron nanowire α-c [001] is distinct from that of the bulk boron and other under-considered boron nanowires. In addition, the electronic properties of these considered boron nanowires obtained from the unit cell of the bulk α-B are also direction-dependent. Thus, these results of direction dependence of the electronic and magnetic properties of boron nanowires would be reflected on the photoelectronic properties of these materials and bring them to have many promising applications that are novel for the bulk boron.

## Conclusions

In summary, we have performed a systematic study of the stability and electronic and magnetic properties of boron nanowires using the spin-polarized density functional calculations and found that the considered boron nanowires possess the direction dependence of ferromagnetic and semiconducting behaviors, which are distinctly different from those of the boron bulk that is metallic and not magnetic. The physical origins of ferromagnetic and semiconducting properties of boron nanowires were pursued and attributed to the unique surface structures of boron nanowires. Thus, these theoretical findings seem to open a window toward the applications of boron nanowires in electronics, optoelectronics, and spin electronics.

## Competing interests

The authors declare that they have no competing interests.

## Authors’ contributions

This work was finished through the collaboration of all authors. JLL carried out the calculation, analyzed the calculated data, and drafted the manuscript. TH helped analyze the data and participated in revising the manuscript. GWY supervised the work and finalized the manuscript. All authors read and approved the final manuscript.
